# *Streptococcus pneumoniae *early response genes to human lung epithelial cells

**DOI:** 10.1186/1756-0500-1-64

**Published:** 2008-08-12

**Authors:** Xin-Ming Song, Wayne Connor, Karsten Hokamp, Lorne A Babiuk, Andrew A Potter

**Affiliations:** 1 Vaccine and Infectious Disease Organization (VIDO), University of Saskatchewan, Saskatoon, Saskatchewan, S7N 5E3, Canada; 2 Smurfit Institute of Genetics, Trinity College Dublin, Dublin 2, Ireland; 3 University of Alberta, Edmonton, Alberta, T6G 2J9, Canada

## Abstract

**Background:**

*Streptococcus pneumoniae *infection starts from colonization of the host respiratory tract where interaction with host respiratory tract epithelial cells occurs. To investigate pneumococcal genes that are involved in the early stage of interaction with host epithelial cells, transcriptional responses of an encapsulated pathogenic pneumococcal strain TIGR4 upon exposure to human lung epithelial cells A549 for 0.5 h and 1 h time periods were investigated by using TIGR (JCVI) microarray technology. Gene expression changes were validated by quantitative real-time PCR (qRT-PCR) analysis.

**Findings:**

We observed different transcriptional profiles at two incubation time periods in which most gene expressions were down-regulated at 0.5 h but up-regulated at 1 h. Many genes associated with ribonucleotide biosynthesis were down-regulated at both time points, whereas the genes associated with cell envelope, energy metabolism, transport and protein synthesis were mostly up-regulated at 1 h. Furthermore, these profiles were compared to the transcriptomes of a TIGR4-derived strain in response to human macrophages for the same time periods. We found one set of genes that exhibited similar expression changes upon exposure to both types of host cells, including cell envelope-associated *bgaA *(SP0648) and *nanA *(SP1693), and uncharacterized gene clusters such as SP1677–SP1680 and SP1688–SP1690.

**Conclusion:**

These data indicate that at the early stage of interaction with host epithelial cells, a complex gene regulation and expression change occur in bacteria. Some of them might play an essential role during pathogen-host interactions and for the establishment of infection.

## Findings

### Background

As a major bacterial pathogen, *Streptococcus pneumoniae *infection starts from colonization of the human upper respiratory tract, causing respiratory tract diseases such as pneumonia, bronchitis, otitis media and sinusitis. Under certain circumstances, bacteria invade host cells and evade host immunity, causing systemic infections such as bacteremia, sepsis and meningitis. Therefore, the interaction of *S. pneumoniae *with host respiratory tract epithelial cells is an initial step for infection. Many factors that contribute to the colonization and/or invasion of host epithelial cells have been characterized in *S. pneumoniae *(recently reviewed by: [1-3]). However, it is becoming obvious that multiple factors are involved in this complex process [4].

Microarray-based transcriptome studies have been used in many pathogens, investigating their transcriptional responses to host cells [5]. However, they were rarely performed at an early stage of interaction time period, a stage that might be critical for microbes to establish an infection. This is likely due to the difficulty of obtaining sufficient bacterial RNA from a mixture of bacteria and host cells. In *S. pneumoniae*, transcriptome studies were initiated by Orihuela et al. [6] in which an unencapsulated derivative of TIGR4 was investigated following exposure to human pharyngeal epithelial cells (Detroit 562) for 3 h. By using self-spotted pneumococcal oligonucleotide (oligo) microarrays we have also examined gene expression changes of an encapsulated serotype 3 clinical isolate and one unencapsulated avirulent laboratory strain following incubation with human lung epithelial cells (A549) for 1 h and 3 h, respectively [7]. Nevertheless, a lack of information exists regarding pneumococcal gene expression at an early stage of interaction with host cells. The strain-specific gene regulation features of *S. pneumoniae *[8] also prompted our research interests on other serotype strains.

In this study, we have developed a system which can be used to isolate enough bacterial RNA for microarray analysis from encapsulated pathogenic strains following incubation with A549 cells for a short time period. By using TIGR microarrays, we performed transcriptome studies on an encapsulated wild-type strain TIGR4. This study highlighted the gene transcriptional profiles in *S. pneumoniae *and revealed the potential roles of some target genes during pathogen-host interactions.

## Methods

### Incubation of bacteria and host cells

Culturing and incubation of pneumococcal strain TIGR4 (provided by Dr. Caroline A. Obert, St. Jude Children's Research Hospital) and human lung epithelial cells A549 were performed as previously described [7] with minor modifications. Briefly, bacteria grown to early logarithmic-phase at OD_600 _0.3 were collected by centrifugation, re-suspended in antibiotic-free MEM complete medium supplemented with 1% fetal bovine serum (FBS), and incubated with host cells in T75 flasks at a multiplicity of infection 120:1. After incubation, non-adherent bacteria were removed by washing 3 times with 5 ml of antibiotic-free cell culture medium. Host cells were removed by incubation with a host cell lysis buffer containing guanidine thiocyanate (Sigma), β-mercaptoethanol, phenol and ethanol at room temperature for 10 min. Bacterial samples were collected by centrifugation for RNA isolation. Bacteria incubated with cell culture medium for different time points, treated with RNALater (Ambion), were collected as medium control samples.

### Preparation of bacterial RNA

Isolation of bacterial RNA was performed with RiboPure™-Bacteria Kit (Ambion) or a modified method using RNeasy MiniKit (Qiagen) as previously described [7]. From each flask of cell infection, about 2~4 μg bacterial total RNA with less than 10% of eukaryotic RNA contamination could be generated. Medium control RNA samples at each incubation time point were generated by pooling RNAs isolated from 3 separate assays. Genomic DNA contamination was removed by the treatment with RNase-free of DNase I (Ambion).

### Microarray experiment and analysis

TIGR (J. Craig Venter Institute) *S. pneumoniae *70-mer oligo microarray (version 6), provided by the Pathogen Functional Genomics Resource Center (PFGRC), was used in this study. The cDNA synthesis, Cy-dye labelling, and microarray hybridization were carried out according to TIGR's standard operating procedures (SOPs) . Hybridization signals were captured with a GenePix 4200A scanner (Axon Instruments) and the data were processed and analyzed through ArrayPipe [9]. This includes flagging of marker spots, background correction, printTip Loess normalization with Limma, and statistical analysis with Limma's eBayes moderated *t*-test [10]. Gene expressions of fold change ≥ 2.0 (bacteria incubated with host cells vs. bacteria incubated with media) with statistical significance (*p *≤ 0.05) were classified as being significantly changed. In this study, eight independent hybridizations, including four labelled in dye flips, using RNA samples isolated from eight separate assays were performed for each incubation time point.

### Quantitative real-time PCR (qRT-PCR) analysis

The oligo primers used for qRT-PCR analysis (Table 1) were designed from *S. pneumoniae *TIGR4 genome sequences by using Clone Manager Suite 7 (Scientific & Educational Software) and synthesized by Invitrogen. The qRT-PCR reaction and analysis were performed as previously described [7]. For each gene, duplicate reactions were performed on the RNA samples isolated from at least two separate assays for each incubation time point.

**Table 1:** Oligonucleotide primers used for qRT-PCR analysis

**Gene name**	**TIGR4 genome acc. No**.	**Oligonucleotide primers 5' to 3'**	**Amplified product (bp)**
*purH*	SP0050	Sense: TCAAGCAACCAATGCGTTACGGTGAG	110
		Anti-sense: TTTCCCGTTGAGCTGTTTGGCTGAAG	
*strH*	SP0057	Sense: GTGTCAGCCCAAGCAGCTACCATACCAC	128
		Anti-sense: GGCCAAGGCTGGTACAATCTCGATCAGG	
*cbpI*	SP0069	Sense: GCTATGAAGACAGGCTGGTACAAG	133
		Anti-sense: TCACAGCCAAAGCTCCTGAAC	
*nrdD*	SP0202	Sense: TGCAACCAAGCGGATGTATCCAGACG	99
		Anti-sense: TGAAGGAAAGAACGGCAGCCCATAGG	
	SP0287	Sense: CAGTCGGTGCCATTGCAGGTACTTCAAAC	103
		Anti-sense: GCTACAACCAAGGCTGTCAAACCAGTACG	
*caps4A*	SP0346	Sense: GTCAGAGTATCCAGACTACGCATCGAAG	159
		Anti-sense: TCTGATCGCGACACCGAACTAATAGG	
*bgaA*	SP0648^a^	Sense: CAAGCCAGCCGTGAACGCTATAAGG	128
		Anti-sense: GAGTGGGCAGTCAGGGTGAATTTCC	
*gyrA*	SP1219^a^	Sense: GTGCTGCCGCTCAACGTTATACCGAGG	142
		Anti-sense: AAACGCGCTGGCAAGACCAAGGGTTCC	
*pyrR*	SP1278	Sense: GACAGACCGCGAAGTTATCTTGGTGG	115
		Anti-sense: AACTGCTAAACTCACACGCGCAGGAC	
	SP1679	Sense: GGACAGGGGATTACAGTTGATGAGATGG	149
		Anti-sense: GCAGTTGCAGCTACCCTACTTAAGATCG	
	SP1680	Sense: GCCTGCATAACCATTTGGCTGATGTG	127
		Anti-sense: AGCATTCGACGAAGCGAGTGACATTG	
	SP1688	Sense: AAGTGAACGAAGGGCTACTGCTACTGTC	136
		Anti-sense: GCTACCGATTGTAGCACCAGGTATTG	
*nanA*	SP1693	Sense: GACATATTCGAAAGCGGGCGTAACGG	117
		Anti-sense: GCGTTCATCTGCACCTGCGATCAAAG	
*purR*	SP1979^a^	Sense: AGGCAGCCGTGTCTTGATTGTGG	120
		Anti-sense: TTGTCCGCAAAGACCGCTACACC	

^a. ^Obtained from [7].

## Results and discussion

### Transcriptional responses of *S. pneumoniae *to host epithelial cells

**Figure 1:**
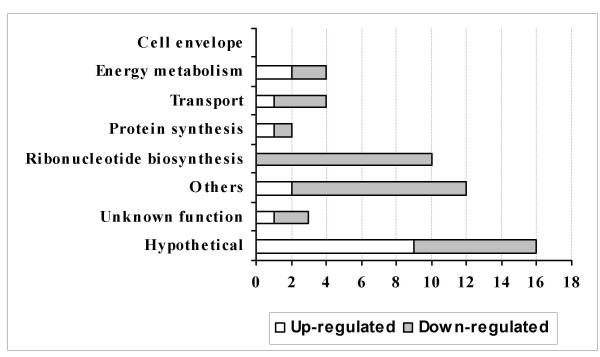
**Transcriptional profiles of functional categories of genes identified in microarray analysis at 0.5 h incubation time period**. The number of differentially regulated genes (*x*-axis) identified in microarray analysis in *S. pneumoniae *TIGR4 following incubation with A549 cells for 0.5 h time period. They are represented in different functional categories (*y*-axis) and marked with up-regulated (open bars) and down-regulated (grey bars) expressions. No cell envelope genes were identified.

**Figure 2:**
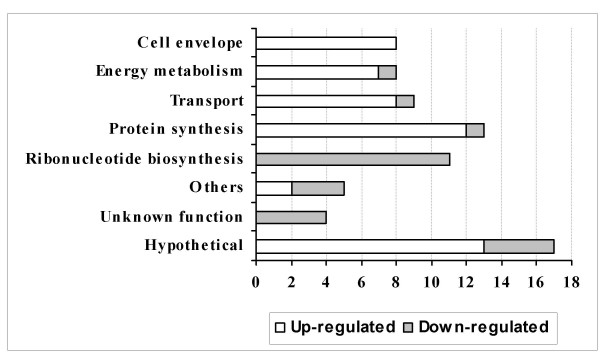
**Transcriptional profiles of functional categories of genes identified in microarray analysis at 1 h incubation time period**. The number of differentially regulated genes (*x*-axis) identified in microarray analysis in *S. pneumoniae *TIGR4 following incubation with A549 cells for 1 h time period. They are represented in different functional categories (*y*-axis) and marked with up-regulated (open bars) and down-regulated (grey bars) expressions.

**Figure 3:**
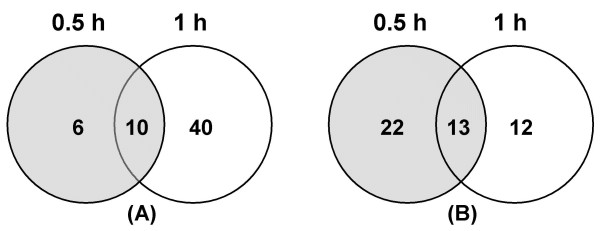
**Venn diagrams of microarray identified genes**. The up-regulated (A) and down-regulated (B) genes in *S. pneumoniae *TIGR4 following incubation with A549 cells for 0.5 h (grey circles) and 1 h (open circles), respectively.

Microarray analysis revealed many gene expression changes following exposure to A549 cells (Table 2). At 0.5 h, most gene expressions were down-regulated (35 vs. 16) and a smaller number of genes changed (Fig. 1). At 1 h, more genes were changed and most of them were up-regulated (50 vs. 25) (Fig. 2). Furthermore, most of those changed genes were only defined at a certain incubation time period (Fig. 3). These data indicate divergent transcriptional profiles between 0.5 h and 1 h incubation time periods. Repressed transcriptional profiles at 0.5 h (Fig. 1) suggest that the interaction with human respiratory tract epithelial cells, a natural reservoir for *S. pneumoniae*, might be a favourable situation for pneumococci. This is in contrast to the *S. pneumoniae *transcriptomes to macrophages, where most genes that showed transcriptional changes at the early stage of interactions were up-regulated (Song XM, Connor W, Hokamp K, Babiuk LA, Potter AA: Transcriptome studies on *Streptococcus pneumoniae*, illustration of early response genes to THP-1 human macrophages, submitted). When incubated for 1 h, bacterial survival, growth and virulence mechanisms appear to be activated, apparent from an induced expression of genes in cell envelope, energy metabolism, transport, protein synthesis, and hypothetical proteins (Fig. 2).

**Table 2:** Microarray identified genes in pneumococcal strain TIGR4 upon exposure to A549 cells for 0.5 h and 1 h time periods

**Function/gene name**	**Protein**	**TIGR4 genome acc. No**.	**Incubation time**	
			
			**0.5 h**	**1 h**
**Cell envelope**				
*cbpI *^a^	choline binding protein I	SP0069		2.8
*cps4A*	capsular polysaccharide biosynthesis protein Cps4A	SP0346		2.9
*cps4B*	capsular polysaccharide biosynthesis protein Cps4B	SP0347		2.0
*cps4C*	capsular polysaccharide biosynthesis protein Cps4C	SP0348		3.3
*cps4E*	capsular polysaccharide biosynthesis protein Cps4E	SP0350		2.9
*cps4I*	UDP-N-acetylglucosamine-2-epimerase	SP0357		2.4
*bgaA*	β-galactosidase	SP0648		17.0
*nanA *^a^	neuraminidase A, authentic frameshift	SP1693		16.5
				
**Energy metabolism**				
*agaS*	sugar isomerase domain protein AgaS	SP0065		5.6
*pyk*	pyruvate kinase	SP0897	-2.7	
*glgA*	glycogen synthase	SP1124		3.8
	acetoin dehydrogenase complex, E2 component, dihydrolipoamide acetyltransferase, putative	SP1162		2.7
*zwf*	glucose-6-phosphate 1-dehydrogenase	SP1243	-2.7	
*scrB*	sucrose-6-phosphate hydrolase	SP1724	3.0	4.4
*galT*	galactose-1-phosphate uridylyltransferase	SP1852		2.7
*galK*	galactokinase	SP1853		2.3
*recP*	transketolase	SP2030		-3.6
*arcA*	arginine deiminase	SP2148	4.6	
*gplK*	glycerol kinase	SP2186		3.0
				
**Hypothetical proteins**				
	conserved hypothetical protein	SP0024		-2.6
	hypothetical protein	SP0026	-2.3	
	hypothetical protein	SP0052	-3.5	-5.6
	hypothetical protein	SP0067	2.4	2.1
	conserved hypothetical protein	SP0095	-2.4	
	conserved hypothetical protein	SP0159	-2.3	
	hypothetical protein	SP0190	2.3	
	hypothetical protein	SP0203	-2.5	
	conserved hypothetical protein	SP0207	-2.1	
	conserved hypothetical protein	SP0288	-4.2	-2.2
	conserved hypothetical protein	SP0742		-2.9
	conserved hypothetical protein	SP0951		2.4
	conserved hypothetical protein	SP1003		2.1
	hypothetical protein	SP1049	2.0	
	hypothetical protein	SP1059		4.4
	conserved hypothetical protein	SP1174		2.4
	hypothetical protein	SP1198	2.7	2.6
	hypothetical protein	SP1199	2.9	2.0
	conserved hypothetical protein	SP1601		2.4
	hypothetical protein	SP1677		10.3
	hypothetical protein	SP1678	2.9	6.1
	hypothetical protein	SP1679	4.6	9.6
	conserved hypothetical protein	SP1680	5.3	11.5
	hypothetical protein	SP2183	2.7	4.1
				
**Others**				
	bacteriocin, putative	SP0109	2.3	
	lactose phosphotransferase system repressor, degenerate	SP0169		2.2
	dihydropteroate synthase	SP0289	-2.2	-2.1
*acpP*	acyl carrier protein	SP0418	-2.0	
*fabF*	3-oxoacyl-(acyl-carrier-protein) synthase II	SP0422	-2.4	
*accD*	acetyl-CoA carboxylase, carboxyl transferase subunit beta	SP0426	-2.4	
*accA*	acetyl-CoA carboxylase, carboxyl transferase subunit alpha	SP0427	-3.4	
*ilvB*	acetolactate synthase, large subunit, biosynthetic type	SP0445	-2.8	
*zmpB*	zinc metalloprotease ZmpB	SP0664	-2.1	
*ilvE*	branched-chain amino acid aminotransferase	SP0856	-2.0	
	preprotein translocase, SecG subunit, putative	SP0974		2.5
*asd*	aspartate-semialdehyde dehydrogenase	SP1013	-2.0	
*bta*	bacterocin transport accessory protein	SP1499	-2.7	-2.4
	transcriptional regulator, MerR family	SP1856	2.0	
*groEL*	chaperonin, 60 kDa	SP1906		-2.4
				
**Protein synthesis**				
*rpsD*	ribosomal protein S4	SP0085		2.7
*rpsJ*	ribosomal protein S10	SP0208		4.1
*rplW*	ribosomal protein L23	SP0211		2.9
*rpsC*	ribosomal protein S3	SP0215		2.0
*infA*	translation initiation factor IF-1	SP0232		2.4
*valS*	valyl-tRNA synthetase	SP0568	-2.1	
*rplK*	ribosomal protein L11	SP0630		2.5
*infC*	translation initiation factor IF-3	SP0959		2.5
*rpml*	ribosomal protein L35	SP0960		3.9
*rpsR*	ribosomal protein S18	SP1539		2.8
*rpsF*	ribosomal protein S6	SP1541	2.9	3.0
*rpmH*	ribosomal protein L34	SP1993		2.4
*rpmG*	ribosomal protein L33	SP2135		2.1
*yfiA*	ribosomal subunit interface protein	SP2206		-3.9
				
**Purine and pyrimidine ribonucleotide biosynthesis**				
*purA*	adenylosuccinate synthetase	SP0019	-2.5	
*purC *^a^	phosphoribosylaminoimidazole-succinocarboxamide synthase	SP0044	-5.1	-4.7
*purH*	phosphoribosylaminoimidazolecarboxamide formyltransferase-IMP cyclohydrolase	SP0050	-15.3	-4.1
*purE *^a^	phosphoribosylaminoimidazole carboxylase, catalytic subunit	SP0053	-6.4	-8.5
*purK *^a^	phosphoribosylaminoimidazole carboxylase, ATPase subunit	SP0054		-2.4
*nrdD*	anaerobic ribonucleoside-triphosphate reductase	SP0202	-4.4	-4.3
*nrdG*	anaerobic ribonucleoside-triphosphate reductase activating protein	SP0205	-3.4	-2.8
*thyA*	thymidylate synthase	SP0669	-2.2	
*pyrK*	dihydroorotate dehydrogenase, electron transfer subunit	SP0963		-3.6
*nrdH*	NrdH-redoxin	SP1178		-2.1
*carB*	carbamoyl-phosphate synthase, large subunit	SP1275		-4.2
*pyrR*	pyrimidine operon regulatory protein	SP1278	-2.1	-7.8
*guaA *^a^	GMP synthase	SP1445	-2.4	
*purR*	pur operon repressor	SP1979	-2.7	-2.3
				
**Transport**				
	PTS system, IIA component	SP0064	2.2	3.4
	PTS system, mannose-specific IID component	SP0282	-3.7	
	xanthine-uracil permease family protein	SP0287	-8.4	-5.3
	O-antigen transporter RfbX, putative	SP0356		2.5
	PTS system, IIC component, putative	SP0647		4.3
	sugar ABC transporter, ATP-binding protein	SP0846	-2.1	
	ABC transporter, permease protein	SP1688		5.3
	ABC transporter, permease protein	SP1689		2.8
	ABC transporter, substrate-binding protein	SP1690		2.1
*msmE*	sugar ABC transporter, sugar-binding protein	SP1897		2.1
*malD*	maltodextrin ABC transporter, permease protein	SP2110		2.6
				
**Unknown function**				
	vanZ protein, putative	SP0049	-2.9	
	ACT domain protein	SP0238	-2.1	-3.4
	HIT family protein	SP0521		-2.4
*gid*	Gid protein	SP0943		-2.2
	flavoprotein	SP1231		-2.0
*usp45*	secreted 45 kd protein	SP2216	2.1	

^a ^genes that are also involved in pathogenesis according to TIGR genome annotation

We also observed a common change between two incubation time points, that more than 10 purine and pyrimidine ribonucleotide biosynthesis genes, including purine and pyrimidine regulatory genes *purR *and *pyrR*, were consistently down-regulated (Table 2; Figs. 1, 2). The roles of ribonucleotide biosynthesis and their gene regulation mechanism in *S. pneumoniae *are largely unknown. However, down-regulation of these genes in pneumococci appears to occur only at an early stage of interaction with host epithelial cells, but not at 3 h [6,7]. It also might be specific to the pneumococcal strains and the types of host cells because most of those ribonucleotide biosynthesis genes were unchanged in a serotype 3 strain [7] or when the TIGR4-derived strain was exposed to the host macrophages (Song XM, Connor W, Hokamp K, Babiuk LA, Potter AA: Transcriptome studies on *Streptococcus pneumoniae*, illustration of early response genes to THP-1 human macrophages, submitted). Perhaps this is the shift of bacteria to parasitism enabling the uptake of substrates from the host cells [11], or the indication of metabolic changes in different pneumococcal strains in different host environment.

Microarray data have been deposited in the ArrayExpress microarray database  under accession No. E-FPMI-15.

### Microarray data validation

**Figure 4:**
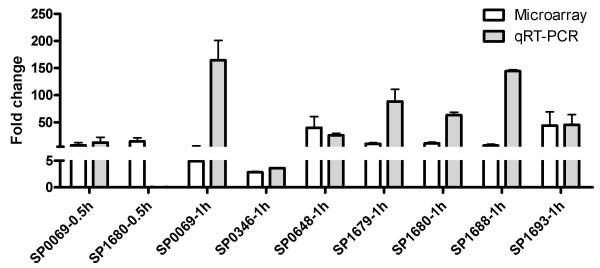
**Validation of up-regulated genes by qRT-PCR**. The up-regulated genes identified in microarray (open bars) and qRT-PCR (grey bars) analyses. The characterized genes incubated with A549 cells for different time periods (0.5 h or 1 h) are marked on the *x*-axis. For consistency, each gene is indicated by the TIGR4 genome accession number (SP), not the gene name. The fold changes (mean) from all the repeated assays with standard deviations are marked on the *y*-axis. Scales on the *y*-axis (0~5, 5~250) are not continuous due to large changes for some genes.

**Figure 5:**
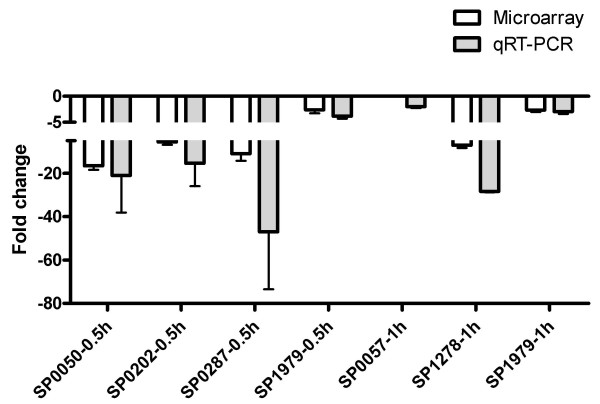
**Validation of down-regulated genes by qRT-PCR**. The down-regulated genes identified in microarray (open bars) and qRT-PCR (grey bars) analyses. The characterized genes incubated with A549 cells for different time periods (0.5 h or 1 h) are marked on the *x*-axis. For consistency, each gene is indicated by the TIGR4 genome accession number (SP), not the gene name. The fold changes (mean) from all the repeated assays with standard deviations are marked on the *y*-axis. Scales on the *y*-axis (0~-5, -5~-80) are not continuous due to large changes for some genes.

To confirm gene expression changes identified in microarray analysis, we performed qRT-PCR analysis on 16 selected genes at different incubation time point, most of them associated with cell envelope, ribonucleotide biosynthesis, SP1677-SP1680 and SP1688-SP1690 gene clusters. Except for the unchanged SP1680 at 0.5 h, all the other gene expressions changed in accordance to the microarray data, but at a greater average fold change in the qRT-PCR analysis (Figs. 4, 5). Expression change of SP0057 at 1 h was only obtained from qRT-PCR assay because the strain-specific oligo probes were absent on the microarrays (Fig. 5).

### Common response genes to host cells

In a separate transcriptome study, we have investigated gene expression changes of a TIGR4-derived unencapsulated strain following incubation with human THP-1 derived macrophages for different time points (0.5 h, 1 h and 3 h) (Song XM, Connor W, Hokamp K, Babiuk LA, Potter AA: Transcriptome studies on *Streptococcus pneumoniae*, illustration of early response genes to THP-1 human macrophages, submitted). As similar experimental procedures and microarray technology were applied, we compared these two studies and revealed many common response genes at early interaction time periods, including well characterized virulence genes such as *bgaA *and *nanA*, and uncharacterized gene clusters such as SP1677-SP1680 (hypothetical) and SP1688-SP1690 (ABC transporter) (Table 3). It indicates common features in pneumococcal gene responses to different types of host cells. Although the interactions with host epithelial cells and macrophages are mainly associated with different pathogenesis processes, reflected by the colonization of host epithelial cells and the survival from host phagocytic cells, we assume these processes are closely related and some of those genes might be assigned with multiple functions.

**Table 3:** Common response genes to both A549 cells and THP-1 derived macrophages at 0.5 h and 1 h incubation time periods

**Function/gene name**	**Protein**	**TIGR4 genome acc. No**.	**A549^a^**		**THP-1^b^**	
			
			**0.5 h**	**1 h**	**0.5 h**	**1 h**
**Cell envelope**						
*cbpI*^c^	choline binding protein I	SP0069		2.8		8.4
*bgaA*	beta-galactosidase	SP0648		17.0	3.4	26.9
*nanA*^c^	neuraminidase A, authentic frameshift	SP1693		16.5	3.9	47.1
						
**Energy metabolism**						
*agaS*	sugar isomerase domain protein AgaS	SP0065		5.6		10.3
*glgA*	glycogen synthase	SP1124		3.8		5.4
	acetoin dehydrogenase complex, E2 component, dihydrolipoamide acetyltransferase, putative	SP1162		2.7	4.9	6.0
*scrB*	sucrose-6-phosphate hydrolase	SP1724	3.0	4.4	2.4	4.7
*galT*	galactose-1-phosphate uridylyltransferase	SP1852		2.7	2.6	4.4
*galK*	galactokinase	SP1853		2.3	2.3	2.8
*recP*	transketolase	SP2030		-3.6	-2.0	-2.5
*gplK*	glycerol kinase	SP2186		3.0		4.1
						
**Hypothetical proteins**						
	hypothetical protein	SP0052	-3.5	-5.6	-2.6	-3.5
	hypothetical protein	SP0067	2.4	2.1		4.1
	conserved hypothetical protein	SP0159	-2.3		-2.0	
	conserved hypothetical protein	SP0742		-2.9	-6.5	-3.2
	conserved hypothetical protein	SP1003		2.1	2.1	3.4
	hypothetical protein	SP1059		4.4	56.3	16.0
	conserved hypothetical protein	SP1174		2.4	2.7	4.4
	hypothetical protein	SP1198	2.7	2.6		2.8
	hypothetical protein	SP1199	2.9	2.0		2.2
	hypothetical protein	SP1677		10.3		14.6
	hypothetical protein	SP1678	2.9	6.1		6.9
	hypothetical protein	SP1679	4.6	9.6		6.6
	conserved hypothetical protein	SP1680	5.3	11.5	2.0	14.6
						
**Others**						
	lactose phosphotransferase system repressor, degenerate	SP0169		2.2	15.4	6.0
*acpP*	acyl carrier protein	SP0418	-2.0		-2.3	
*fabF*	3-oxoacyl-(acyl-carrier-protein) synthase II	SP0422	-2.4			-5.1
*bta*	bacterocin transport accessory protein	SP1499	-2.7	-2.4	-4.2	-2.2
						
**Protein synthesis**						
*rpsD*	ribosomal protein S4	SP0085		2.7		2.4
*rpsJ*	ribosomal protein S10	SP0208		4.1		2.9
*rpsC*	ribosomal protein S3	SP0215		2.0	2.2	
*infC*	translation initiation factor IF-3	SP0959		2.5	2.2	2.3
*rpmI*	ribosomal protein L35	SP0960		3.9	2.2	
*rpsF*	ribosomal protein S6	SP1541	2.9	3.0		2.2
*yfiA*	ribosomal subunit interface protein	SP2206		-3.9	-2.6	-2.5
						
**Purine and pyrimidine ribonucleotide biosynthesis**						
*purC*^c^	phosphoribosylaminoimidazole-succinocarboxamide synthase	SP0044	-5.1	-4.7	-2.4	-7.8
*purH*	phosphoribosylaminoimidazolecarboxamide formyltransferase-IMP cyclohydrolase	SP0050	-15.3	-4.1	-4.3	-5.5
*purE*^c^	phosphoribosylaminoimidazole carboxylase, catalytic subunit	SP0053	-6.4	-8.5		-4.2
*carB*	carbamoyl-phosphate synthase, large subunit	SP1275		-4.2		-2.6
*pyrR*	pyrimidine operon regulatory protein	SP1278	-2.1	-7.8	-2.3	-4.4
						
**Transport**						
	PTS system, IIA component	SP0064	2.2	3.4		6.5
	PTS system, mannose-specific IID component	SP0282	-3.7		-2.4	
	xanthine-uracil permease family protein	SP0287	-8.4	-5.3		-2.3
	PTS system, IIC component, putative	SP0647		4.3	3.5	8.2
	ABC transporter, permease protein	SP1688		5.3	2.4	13.6
	ABC transporter, permease protein	SP1689		2.8	3.7	18.9
	ABC transporter, substrate-binding protein	SP1690		2.1	3.9	21.6
*msmE*	sugar ABC transporter, sugar-binding protein	SP1897		2.1		4.1
*malD*	maltodextrin ABC transporter, permease protein	SP2110		2.6	2.1	6.4
						
**Unknown function**						
	vanZ protein, putative	SP0049	-2.9		-3.4	-4.9
	ACT domain protein	SP0238	-2.1	-3.4		-2.3
	HIT family protein	SP0521		-2.4		-2.1
	flavoprotein	SP1231		-2.0	-2.3	

^a ^Genes identified in this study.

^b ^Genes identified in Song XM, Connor W, Hokamp K, Babiuk LA, Potter AA: Transcriptome studies on *Streptococcus pneumoniae*, illustration of early response genes to THP-1 human macrophages, submitted.

^c ^Genes that are also involved in pathogenesis according to TIGR genome annotation.

### The exoglycosidase family genes

In *S. pneumoniae*, the *bgaA*-encoded β-galactosidase (BgaA) and the *nanA*-encoded neuraminidase (NanA) belong to a family of exoglycosidases exposed on the bacterial surface. Studies have demonstrated that both enzymes, especially NanA, are involved in adherence to host respiratory tract epithelial cells, possibly by clearing host cell surface structures and secreted components to enhance pathogen-host interactions [12-15]. Recently, it was demonstrated that BgaA and NanA, together with StrH (β-*N*-aceylglucosaminidase), act sequentially to remove sialic acid, galactose and *N*-acetylglucosamine [15]. These reports demonstrated the importance of *S. pneumoniae *to deglycosylate human targets during colonization and/or pathogenesis.

In this study, expression of *bgaA *(SP0648) and *nanA *(SP1693) was highly induced when incubated with A549 cells for 1 h in both microarray and qRT-PCR analyses (Table 2; Fig. 4). Further qRT-PCR assay revealed an unchanged expression of *strH *(SP0057) (Fig. 5), correlated to the previous observation that StrH was not involved in the adherence [15]. The enhanced expression of *bgaA *and *nanA *was also observed in a TIGR4-derived strain when exposed to human macrophages for 0.5 h and 1 h time periods (Table 3). It suggests that both *bgaA *and *nanA *belong to a family of conserved early response genes. Clearing host cell surface components and accessing to the host cells are a priority for bacteria at the early stage of pathogen-host interactions.

### Other genes

The *cbpI *(SP0069), encoding choline binding protein I, was also up-regulated in expression (Table 2; Fig. 4). The choline binding proteins (CBPs) are a family of surface proteins, many of them are involved in colonization of nasopharynx [16]. However, *cbpI *was the only CBP gene that was identified in this study. The function of CbpI is still unclear but its crystal structure has been solved [17]. Whether it is important in colonization, most CBPs might not be required at the early stage of interaction with host epithelial cells.

Because of strain-specific gene regulations in *S. pneumoniae *[7,8], different microarray technologies and experimental conditions, some potential gene targets might be missed in our transcriptome studies. For example, the *pspC *(SP1417) gene was reported to be up-regulated in a serotype 2 strain D39 within 1 h post-infection in mice [18]. However, expression change of *pspC *was not identified in our assays, despite of a degenerated PspC carried by the TIGR4 genome (TIGR). Another unchanged gene cluster was the *rlrA *pathogenicity islet genes (SP0461-SP0468) encoding pneumococcal pili [19,20]. All of these TIGR4-specific oligo probes were carried by the TIGR microarrays, and they were clearly identified in our studies of the regulation mechanisms for the pilus locus genes (Song XM, Connor W, Hokamp K, Babiuk LA, Potter AA: The growth phase-dependent regulation of the pilus locus genes by two-component system TCS08 in *Streptococcus pneumoniae*, submitted). We could therefore exclude the technical concern for these genes in our microarray analysis. Earlier studies suggested that pneumococcal pili were mainly involved in the host cell adhesion [21]. Recently, Rosch, et al. defined the restricted functions of pili in invasion of host lung epithelial cells [22], suggesting its roles at a late stage of pathogen-host interactions. If this is the case, also supported by our negative findings, the *rlrA *pilus locus genes are not likely to be involved in the early stage of interaction with host epithelial cells.

## Conclusion

The data presented here provide the first assessment of *S. pneumoniae *early response genes to human lung epithelial cells. It revealed gene expression changes that might be associated with bacterial adaptation, survival, growth and colonization. Up-regulation of several cell envelope genes, such as *bgaA *and *nanA*, and the genes with unknown functions, is likely required for a successful colonization. The specific roles of the identified genes and the functions of coordinated regulation of multiple genes have yet to be further investigated.

## Competing interests

The authors declare that they have no competing interests.

## Authors' contributions

XS designed project, obtained funding support, performed microarray and qRT-PCR assays, data analysis, manuscript preparation and editing. WC contributed to the microarray experimental assays and RNA isolation. KH provided critical support for the microarray data analysis and MIAME compliance. LAB and AAP provided funding support and participated discussions and manuscript preparation. All authors have read and approved the final manuscript.

## References

[B1] Bergmann S, Hammerschmidt S (2006). Versatility of pneumococcal surface proteins. Microbiology.

[B2] Hammerschmidt S (2006). Adherence molecules of pathogenic pneumococci. Curr Opin Microbiol.

[B3] Mitchell TJ (2006). *Streptococcus pneumoniae *: infection, inflammation and disease. Adv Exp Med Biol.

[B4] Hammerschmidt S, Wolff S, Hocke A, Rosseau S, Muller E, Rohde M (2005). Illustration of pneumococcal polysaccharide capsule during adherence and invasion of epithelial cells. Infect Immun.

[B5] Waddell SJ, Butcher PD, Stoker NG (2007). RNA profiling in host-pathogen interactions. Curr Opin Microbiol.

[B6] Orihuela CJ, Radin JN, Sublett JE, Gao G, Kaushal D, Tuomanen EI (2004). Microarray analysis of pneumococcal gene expression during invasive disease. Infect Immun.

[B7] Song XM, Connor W, Jalal S, Hokamp K, Potter AA (2008). Microarray analysis of *Streptococcus pneumoniae * gene expression changes to human lung epithelial cells. Can J Microbiol.

[B8] Hendriksen WT, Silva N, Bootsma HJ, Blue CE, Paterson GK, Kerr AR, de Jong A, Kuipers OP, Hermans PW, Mitchell TJ (2007). Regulation of gene expression in *Streptococcus pneumoniae * by response regulator 09 is strain dependent. J Bacteriol.

[B9] Hokamp K, Roche FM, Acab M, Rousseau ME, Kuo B, Goode D, Aeschliman D, Bryan J, Babiuk LA, Hancock RE, Brinkman FS (2004). ArrayPipe: a flexible processing pipeline for microarray data. Nucleic Acids Res.

[B10] Smyth GK, Gentleman RCVDSIRHW (2005). Limma: linear models for microarray data.. Bioinformatics and computational biology solutions using R and bioconductor.

[B11] Cecchini KR, Gorton TS, Geary SJ (2007). Transcriptional responses of *Mycoplasma gallisepticum* strain R in association with eukaryotic cells. J Bacteriol.

[B12] Orihuela CJ, Gao G, Francis KP, Yu J, Tuomanen EI (2004). Tissue-specific contributions of pneumococcal virulence factors to pathogenesis. J Infect Dis.

[B13] Manco S, Hernon F, Yesilkaya H, Paton JC, Andrew PW, Kadioglu A (2006). Pneumococcal neuraminidases A and B both have essential roles during infection of the respiratory tract and sepsis. Infect Immun.

[B14] Tong HH, Blue LE, James MA, DeMaria TF (2000). Evaluation of the virulence of a *Streptococcus pneumoniae * neuraminidase-deficient mutant in nasopharyngeal colonization and development of otitis media in the chinchilla model. Infect Immun.

[B15] King SJ, Hippe KR, Weiser JN (2006). Deglycosylation of human glycoconjugates by the sequential activities of exoglycosidases expressed by *Streptococcus pneumoniae *. Mol Microbiol.

[B16] Gosink KK, Mann ER, Guglielmo C, Tuomanen EI, Masure HR (2000). Role of novel choline binding proteins in virulence of *Streptococcus pneumoniae *. Infect Immun.

[B17] Paterson NG, Riboldi-Tunicliffe A, Mitchell TJ, Isaacs NW (2006). Overexpression, purification and crystallization of a choline-binding protein CbpI from *Streptococcus pneumoniae *. Acta Crystallogr Sect F Struct Biol Cryst Commun.

[B18] Quin LR, Moore QC, Thornton JA, McDaniel LS (2008). Peritoneal challenge modulates expression of pneumococcal surface protein C during bacteremia in mice. Infect Immun.

[B19] Nelson AL, Ries J, Bagnoli F, Dahlberg S, Falker S, Rounioja S, Tschop J, Morfeldt E, Ferlenghi I, Hilleringmann M, Holden DW, Rappuoli R, Normark S, Barocchi MA, Henriques-Normark B (2007). RrgA is a pilus-associated adhesin in *Streptococcus pneumoniae *. Mol Microbiol.

[B20] Hilleringmann M, Giusti F, Baudner BC, Masignani V, Covacci A, Rappuoli R, Barocchi MA, Ferlenghi I (2008). Pneumococcal pili are composed of protofilaments exposing adhesive clusters of Rrg A. PLoS Pathog.

[B21] Barocchi MA, Ries J, Zogaj X, Hemsley C, Albiger B, Kanth A, Dahlberg S, Fernebro J, Moschioni M, Masignani V, Hultenby K, Taddei AR, Beiter K, Wartha F, von Euler A, Covacci A, Holden DW, Normark S, Rappuoli R, Henriques-Normark B (2006). A pneumococcal pilus influences virulence and host inflammatory responses. Proc Natl Acad Sci U S A.

[B22] Rosch JW, Mann B, Thornton J, Sublett J, Tuomanen E (2008). Convergence of Regulatory Networks on the Pilus Locus of *Streptococcus pneumoniae *. Infect Immun.

